# Circular RNA circRHOT1 contributes to pathogenesis of non-small cell lung cancer by epigenetically enhancing C-MYC expression through recruiting KAT5

**DOI:** 10.18632/aging.203417

**Published:** 2021-08-18

**Authors:** Xiaoyan Ren, Jiangang Yu, Lili Guo, Hong Ma

**Affiliations:** 1 Department of Anesthesiology, The First Affiliated Hospital of China Medical University, Shenyang, Liaoning Province, China

**Keywords:** non-small cell lung cancer, proliferation, circRHOT1, KAT5, c-MYC, epigenetic regulation

## Abstract

Non-small cell lung cancer (NSCLC) one of the most prevalent and severe malignancies globally and the molecular mechanisms of NSCLC are poor understood, limiting the development of diagnostic biomarkers and targeted therapies. Circular RNAs (circRNAs) have been identified as a sort of critical regulator in cancer progression. In this study, we identities the epigenetic regulation function of circular RNA circRHOT1 in promoting NSCLC cell proliferation. We found that circRHOT1 were elevated in the clinical tumor tissues relative to that in the peritumor tissues from NSCLC patients. circRHOT1 was up-regulated in human lung cancer cell lines compared with normal human lung epithelial cell line. MTT assays revealed that the silencing of circRHOT1 by siRNA suppressed cell viabilities of NSCLC cells. Colony formation and Edu assays confirmed that circRHOT1 knockdown attenuated NSCLC cell proliferation *in vitro*. Meanwhile, the depletion of circRHOT1 induced NSCLC cell apoptosis and cell cycle arrest *in vitro*. Mechanically, the depletion of circRHOT1 remarkably reduced c-MYC mRNA and protein expression in NSCLC cells. Inhibition of circRHOT1 reduced the enrichment of transcription active marker histone H3 lysine 27 acetylation (H3K27ac) and RNA polymerase II on the promoter of c-MYC. RNA pull down analysis showed that circRHOT1 was able to directly interact with acetyltransferase KAT5 in NSCLC cells. In summary, we concluded that circRHOT1 contributed to pathogenesis of NSCLC by epigenetically enhancing c-MYC expression through recruiting KAT5. CircRHOT1 and KAT5 may be used as the potential targets for NSCLC therapy.

## INTRODUCTION

Lung cancer is the most life-threatening cancer type worldwide which caused approximately 1.8 million death every year [1]. Among which, non-small cell lung cancer (NSCLC) including lung adenocarcinoma (LUAD) and lung squamous cell carcinoma (LUSC) accounts for roughly 85% of lung cancer incidence [2]. The pathogenesis of lung cancer involves various risk factors, such as genetic mutations and smoking [2]. The most prevalent genetic alterations in NSCLC were mutations in the epidermal growth factor receptor (EGFR) and Kirsten rat sarcoma (KRAS), which were profoundly involved in the initiation and progression of NSCLC [3]. Hence, these genes are regarded as the primary targets in development of therapeutic agents, such as tyrosine kinase inhibitors (TKIs), over the past decades [4]. However, patients frequently developed to acquired resistance after treatment with TKIs [5, 6]. Hence, researches to develop new therapies are urgent to enlarge the clinical benefit of NSCLC.

Circular RNAs (circRNAs) is a class of noncoding RNAs recently recognized as important participants in cell functions, which is characterized by covalently closed looped RNA sequence [7]. Increasing studies have proved the abnormal expression of circRNAs during the cancer development, and their potential role as biomarkers and therapeutic targets [8]. CircRNAs was initially found to function as oncogenes or tumour suppressors through acting as sponges of miRNAs and subsequently regulating gene expression [8]. Recent studies also revealed the function of circRNAs as a nucleators or component of protein complex. For example, Zhang et al. suggested that circACC1 facilitated the stabilization and activation of AMP kinase via their interaction, which further modulated glycolysis and fatty acid β-oxidation [9]. CircRHOT1 is found to be upregulated in several cancers such as pancreatic cancer, hepatocellular carcinoma, and breast cancer [10, 11], as well as promotes progression of pancreatic cancer through regulating the proliferation, invasion, and apoptosis, possibly via sponging miR-125a-3p [11, 12]. Moreover, circRHOT1 mediates the suppression of propofol on tumorigenesis of NSCLC via affecting miR-326/FOXM1 regulatory axis [13], which presented circRHOT1 as a possible regulator for NSCLC.

Lysine acetyltransferases (KATs) were recently found to harbor therapeutic potential for cancers [14, 15]. Within the multiple KAT family members, the importance of KAT5 is prominent, as silencing of KAT5 could be lethal Gorrini2007, and compiling evidences have revealed KAT5 as a potential therapeutic for several cancers, such as liver cancer and mesothelioma [14, 16, 17]. It is reported that KAT5 is capable of stabilizing the oncogene c-Myc, that is critical for development of several cancers such as breast cancer and anaplastic thyroid cancer (ATC) [18–20].

In our present work, we found an elevated level of circRHOT1 in tumor tissues from NSCLC patients. Further mechanistic analysis revealed that circRHOT1 could recruit KAT5 to the promoter region of c-MYC, and consequently contributed to the pathogenesis of NSCLC. Our work presented circRHOT1 as a novel potential target for therapy of NSCLC.

## MATERIALS AND METHODS

### Patient samples

The tumor tissues and adjacent normal tissues of lung tumor were obtained from the First Affiliated Hospital of China Medical University. All patients are informed and have signed the written consent. All experiments concerned with patient samples were approved by the ethnic committee of the First Affiliated Hospital of China Medical University.

### Cell culture

In this study, we used several normal and lung cancer cell lines as following: normal human lung epithelial cell line BEAS-2B, human bronchial epithelioid cell line 16HBE, human large cell lung cancer cell line H460 and H661, human non-small cell lung cancer cell line H1299 A549, and human lung squamous carcinoma cell line MES-1 and H226. The 16HBE, H460, H661, H1299 and A549 were cultured in RPMI-1640 medium (Hyclone, USA). MES-1 was cultured in MEM medium (Hyclone). BEAS-2B was cultured in BEGM medium (Hyclone). All culture medium contains 10% FBS (Gibco, USA) and 1% penicillin/streptomycin. All cells were purchased from American Type Culture Collection (ATCC, USA) and were placed in 37°C incubator with humidified atmosphere containing 5% CO_2_. The small interfering RNA targeting circRHOT1 (siRHOT1) and KAT5 (siKAT5), KAT5 overexpressing plasmid (pcDNA-KAT5) and the negative controls (NC) were purchased from RiboBio (China), the sequences were as following: siRHOT1, 5′-UGCCCGGGGAGGAACCUGCU-3′; siKAT5, 5′-AGUAGUUGAUGAGAUUGAGGU-3′; NC, 5’-UUCUCCGAACGUGUCACGU-3′. For cell transfection, H1299 and A549 cells were plated in 6-well plates, the mixture of siRNA, pcDNA-KAT5 or negative control with lipofectamine 2000 (Invitrogen) was added into each well in accordance with manufacturer’s protocol, and incubated for 24 hours.

### Quantitative real time PCR (qPCR)

Trizol reagent (Tarkara, China) was utilized for the extraction of RNAs from patient samples and cells under manufacturer’s description. A total amount of 1μg RNA was taken to perform reverse transcription to cDNA, followed by quantification using a SYBR Green Mixture Kit (Thermo, USA). The relative expression of circRHOT1 and c-Myc were normalized to internal control GAPDH or U6, by using the following primers: circRHOT1, sense, 5′-ATCACCATTCCAGCTGATGT-3′ and antisense, 5′-TGCTGTCTTTGTCTGTTCTTTC-3′; RHOT1, sense, 5′-GGGAGGAACCTCTTCTGGA-3′ and antisense, 5′-ATGAAGAAAGACGTGCGGAT-3′; c-Myc, sense, 5′-GGCTCCTGGCAAAAGGTCA-3′, antisense, 5′-CTGCGTAGTTGTGCTGATGT-3′; GAPDH, sense, 5′-ACAACTTTGGTATCGTGGAAGG3′ and antisense, 5′-GCCATCACGCCACAGTTTC-3′.

### MTT

Viability of H1299 and A549 cells was assessed by MTT assay. A total number of 3 × 10^3^ cells were seeded into 96-well plates after indicated treatment in each experiment. After incubation for 24, 48 and 72 hours, 10 μL MTT (5g/L) was added into each well and react for 4 hours. Subsequently, the culture medium was discarded and replaced with 150 μL DMSO in each well. The absorbance values were measured at 490 nm by a microplate reader (PerkinElmer, Germany).

### Colony formation

H1299 and A549 cells transfect with appropriate were planted into 12-well plated with 500 cells in each well after appropriate transfection, and cultured in 37°C incubator for two weeks. After the visible colonies formed, cells were stained with crystal violet diluted in methanol for 20 minutes. The colonies were captured and counted via a microscope (Olympus, Japan).

### EdU assay

5-ethynyl-2′-deoxyuridine (EdU) labeling was performed to detect cells in S phase by an EdU-labeling kit (Beyotime) according to manufacturer’s protocol. Briefly, cells were incubated with 1 × EdU solution for 2 hours, followed by fix with 4% PFA. Cells were then reacted with click solution in the kit, and stained by Hoechst 33342. The fluorescence was detected and captured by a fluorescence microscope (Carl Zeiss, Germany).

### RNA fluorescence *in situ* hybridization (FISH)

We adopted the Fluorescence *In Situ* Hybridization assay with a commercial kit (Beyotime) to check the localization of circRHOT1 in cytoplasm, under manufacturer’s description. The Cy3-tagged circRHOT1, 18S, and U6 were ordered from GenePharma (China). Cells were photographed by the confocal microscope (Leica, Germany).

### Flow cytometry

Apoptosis and cell cycle were detected through flow cytometry (FCM). H1299 and A549 cells were seeded into 6-well plates and subjected to indicated treatment, and collected for further detection. To detect apoptotic cells, H1299 and A549 cells were double-stained with Annexin V/PI apoptosis detection kit (Beyotime) following manufacturer’s description. For cell cycle evaluation, cells were harvested and fixed by ice-cold 70% ethanol at –4°C for one week. Next, the cells were stained by a mixture of PI, RNase and PBS in ice for 30 minutes. The samples were measured by FACS Caliber (BD Bioscience, USA).

### Western blotting

H1299 and A549 cells were homogenized with ice-cold RIPA buffer (Beyotime), quantified, and separated by 10% SDS-PAGE. The proteins were shifted to PVDF membranes (Millipore, USA), and blocked by 5% BSA for 1 hour at room temperature. Subsequently, the bands were soaked in anti-c-Myc primary antibody (1:1000, Abcam, USA) at 4°C overnight, washed with TBST, followed by incubation with HRP-conjugated secondary antibody. The membranes were visualized via ECL (Thermo) in a Gel imaging system.

### Chromatin immunoprecipitation (ChIP) assay

The ChIP assay was performed by using a ChIP assay kit (Invitrogen) in accordance with manufacturer’s instruction. Briefly, the H1299 and A549 cells were collected after appropriate treatment, fixed, and lysed to obtain the genome. The DNAs were fractured to fragments around 200 bp via ultrasonication, and were incubated with anti-KAT5 overnight at 4°C in rotation, followed by incubation with Protein A agarose for 2 hours. Next, the precipitated complex was washed and eluted. The binding of DNA was evaluated by qPCR.

### RNA pull down

CircRHOT1 and its antisense were obtained by *in vitro* transcription with T7 RNA polymerase, and were labeled by biotin using a Biotin Labeling Mix (Promega, USA). The biotin-labeled circRHOT1 was conjugated with beads, and incubated with cell lysis. Subsequently, the precipitated complex was washed and boiled to obtain protein samples. The samples were analyzed by western blotting experiment using an anti-Flag antibody (Abcam, USA).

### RNA immunoprecipitation (RIP)

Cell fixed with 1% formaldehyde for 10 minutes, and lysed with RIPA lysis buffer containing with RNase inhibitor and protease inhibitor (Sigma). The samples were fractured by sonication and centrifuged. The supernatants were collected and incubated with protein A/G beads (Cell signaling technology, USA) for one hour, followed by interaction with antibody against KAT5 for 4 hours. The enrichment of circRHOT1 was measured by qPCR with IgG as control.

### Xenograft and immunohistology

SCID/nude mice aged 5-weeks were purchased from the Vital River Laboratory Animal Technology (China). Cells subjected to indicated transfection (100 μL, 1 × 10^6^ cells/mouse) were subcutaneously injected in the left fat pad of mice (n = 8). The width and length of tumors and body weight were measured every three days for 5 weeks. The calculation of tumor volume follows the formation: 0.5 × width^2^ × length. At last, the mice were succumbed to death and tumors were isolated, weighted, and captured. Tumors were cut into small pieces, fixed in 4% PFA, imbedded with paraffin and cut into 5-μm-thick sections for further HE staining and IHC. For HE staining, the tumor sections were subjected to 5% eosin staining solution and hematoxylin. For IHC staining of Ki-67, tissues sections were processed with antigen retrieval, blocking with 5% BSA, and incubation with antibody against Ki-67 (1:1000, Abcam) and secondary antibody for 1 hour at room temperature. The visualization of stained cells was achieved by 3,3-diaminobenzidine (DAB, Beyotime). TUNEL assay was performed by using a TUNEL apoptosis detection kit (Thermo) following manufacturer’s protocol.

### Statistics

All data in our work are shown as the mean ± standard deviation. The statical difference was determined by SPSS software and defined by a *p* < 0.05. The two-tailed student’s *t* test and on-way ANOVA were performed for comparison between experimental groups.

## RESULTS

### CircRHOT1 is enhanced in clinical NSCLC samples and NSCLC cell lines

**Figure 1:**
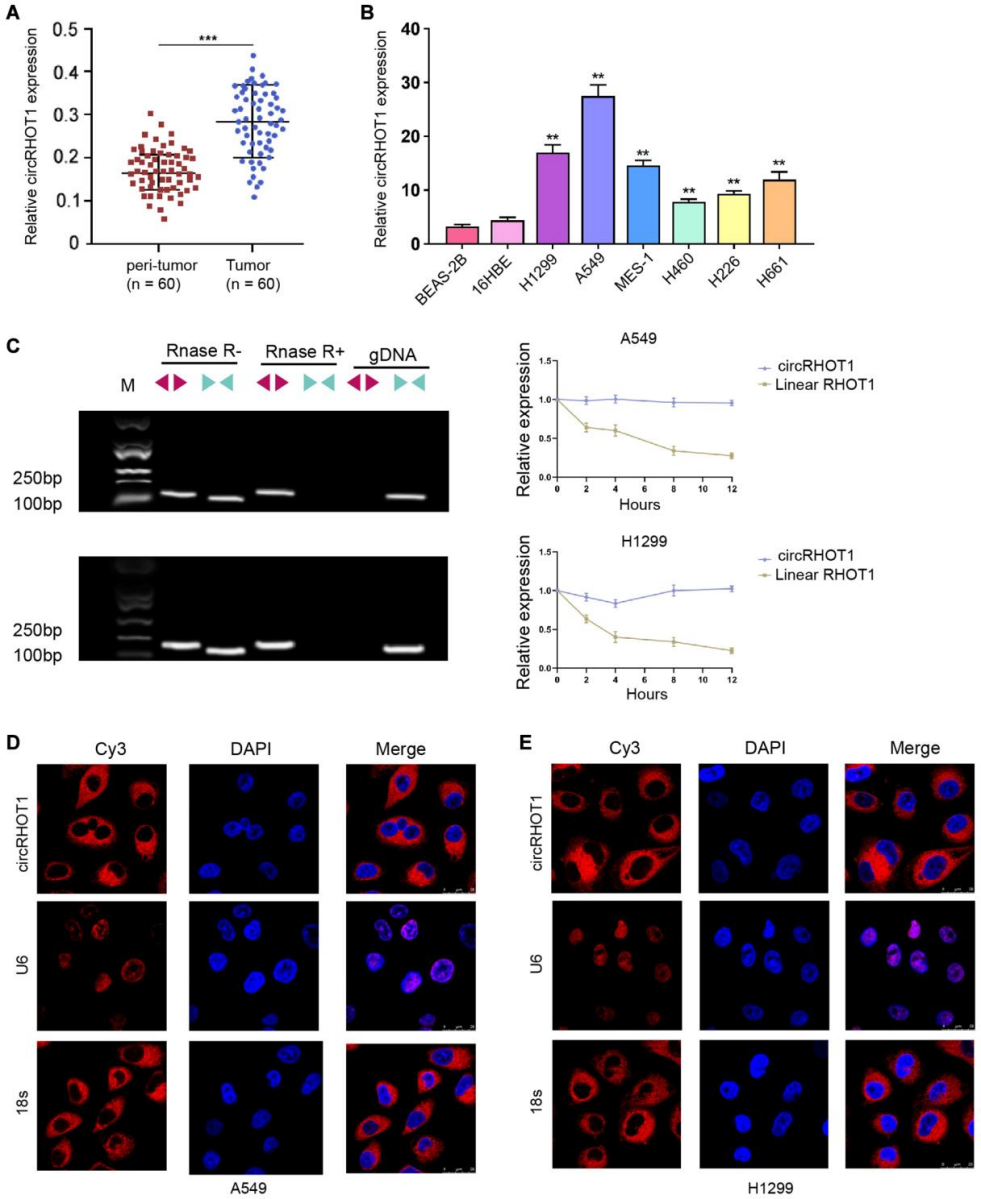
**CircRHOT1 is enhanced in clinical NSCLC samples and NSCLC cell lines.** (**A**) The expression of circRHOT1 was analyzed by qPCR in clinical NSCLC tissues (*n* = 60) and peri-tumor tissues (*n* = 60). (**B**) The expression of circRHOT1 was measured by qPCR in the indicated cells. (**C**) The back-spliced and canonical forms of RHOT1 expression was measured by agarose gel electrophoresis assays and PCR in HCT-116 and HT-29 cells in the presence or absence of RNase R supplementation. (**D** and **E**) The localization of circRHOT1 was determined by FISH analysis and 18s and U6 were the controls. mean ± SD, ^**^P < 0.01.

To confirm the clinical association of circRHOT1 with NSCLC, we detected the expression levels in clinical NSCLC samples. Our data demonstrated that the expression levels of circRHOT1 were elevated in the clinical tumor tissues relative to that in the peritumor tissues from NSCLC patients (Figure 1A). Then, we observed that the expression of circRHOT1 was enhanced in human lung cancer cell lines, including MES-, H226, 16HBE, H460, H661, H1299 and A549 cells, compared with normal human lung epithelial cell line BEAS-2B cell lines (Figure 1B). We identified that H1299 and A549 cells presented higher expression of circRHOT1 in the cell lines, and H1299 and A549 cells were selected to use in the subsequent analysis. We observed that circRHOT1, but not RHOT1, was detectable using divergent primers with the treatment of RNase R (Figure 1C) and the RHOT1 linear form was not observed using convergent primer. FISH assays revealed that circRHOT1 was mainly localized in cytoplasm fraction in H1299 and A549 cells (Figure 1D and 1E).

### The silencing of circRHOT1 attenuates NSCLC cell proliferation *in vitro*

**Figure 2:**
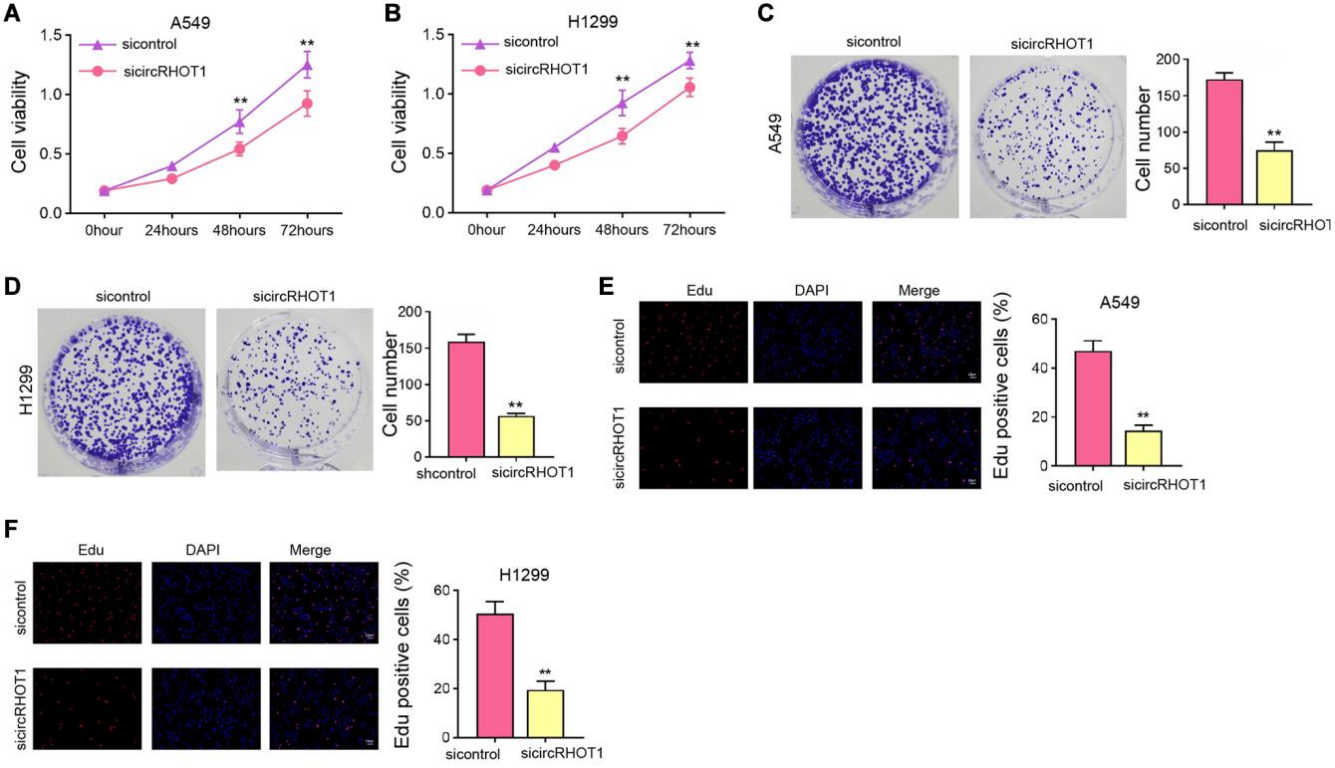
**The silencing of circRHOT1 attenuates NSCLC cell proliferation *in vitro*.** (**A**–**F**) The A549 and H1299 cells were treated with control siRNA or circRHOT1 siRNA. (**A** and **B**) The cell viability was analyzed by MTT assays. (**C** and **D**) The cell proliferation was detected by colony formation assays. (**E** and **F**) The cell survival was detected by Edu assays. mean ± SD, ^**^P < 0.01.

We then evaluated the function of circRHOT1 in mediating NSCLC cell proliferation in H1299 and A549 cells by silencing circRHOT1 expression using siRNA. MTT assays showed that the silencing of circRHOT1 repressed cell viabilities of H1299 and A549 cells (Figure 2A and 2B). Meanwhile, the depletion of circRHOT1 significantly reduced colony formation numbers of H1299 and A549 cells (Figure 2C and 2D). Similarly, the Edu-positive cells were decreased by circRHOT1 knockdown in H1299 and A549 cells (Figure 2E and 2F).

### The depletion of circRHOT1 induces NSCLC cell apoptosis and cell cycle arrest *in vitro*

**Figure 3:**
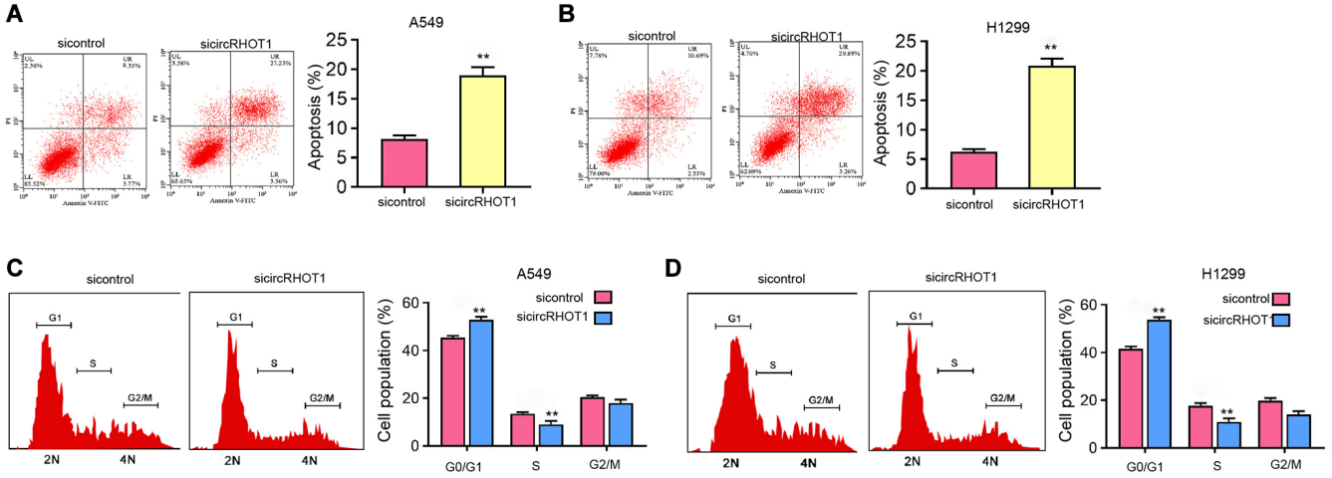
**The depletion of circRHOT1 induces NSCLC cell apoptosis and cell cycle arrest *in vitro*.** (**A**–**D**) The A549 and H1299 cells were treated with control siRNA or circRHOT1 siRNA. (**A** and **B**) The cell apoptosis was measure by flow cytometry analysis. (**C** and **D**) The cell cycle was analyzed by flow cytometry analysis. mean ± SD, ^**^P < 0.01.

We further assessed the effect of circRHOT1 on NSCLC cell apoptosis and cell cycle processes *in vitro*. We found that the silencing of circRHOT1 enhanced the apoptosis of H1299 and A549 cells (Figure 3A and 3B). Moreover, we observed that the proportion of G0/G1 cells was increased while S phase cells were inhibited by the depletion of circRHOT1 in H1299 and A549 cells (Figure 3C and 3D), indicating that circRHOT1 depletion induces NSCLC cell apoptosis and cell cycle arrest *in vitro.*

### CircRHOT1 epigenetically represses c-MYC expression in NSCLC cells

**Figure 4:**
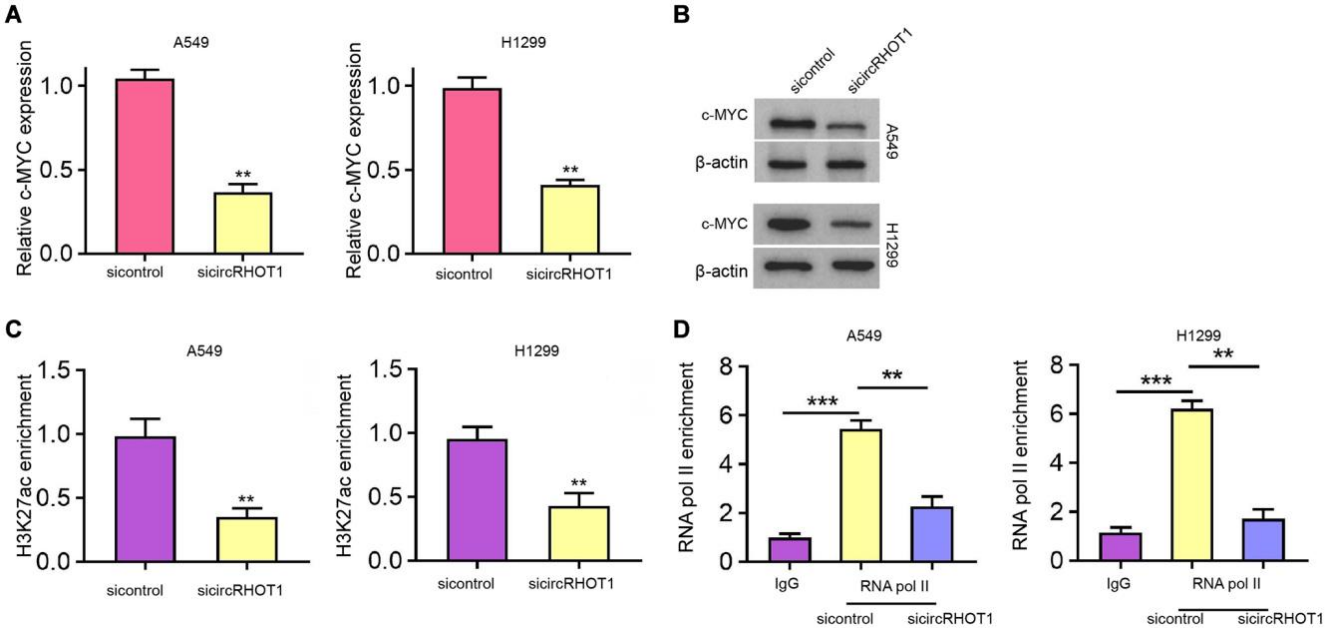
**CircRHOT1 epigenetically represses c-MYC expression in NSCLC cells.** (**A**–**D**) The A549 and H1299 cells were treated with control siRNA or circRHOT1 siRNA. (**A**) The mRNA expression of c-MYC was tested by qPCR. (**B**) The protein levels of c-MYC were determined by Western blot analysis. (**C**) The enrichment of H3K27ac on c-MYC promoter was analyzed by ChIP. (**D**) The enrichment of RNA polymerase II on c-MYC promoter was analyzed by ChIP. mean ± SD, ^**^P < 0.01.

Mechanical investigation identified that the depletion of circRHOT1 remarkably reduced c-MYC mRNA and protein expression in H1299 and A549 cells (Figure 4A and 4B). Moreover, the silencing of circRHOT1 repressed the enrichment of transcription active marker histone H3 lysine 27 acetylation (H3K27ac) on the c-MYC promoter (Figure 4C). Consistently, the enrichment of RNA polymerase II on the promoter of c-MYC was reduced by circRHOT1 knockdown in H1299 and A549 cells (Figure 4D).

### CircRHOT1 epigenetically regulates c-MYC expression by recruiting KAT5 in NSCLC cells

**Figure 5:**
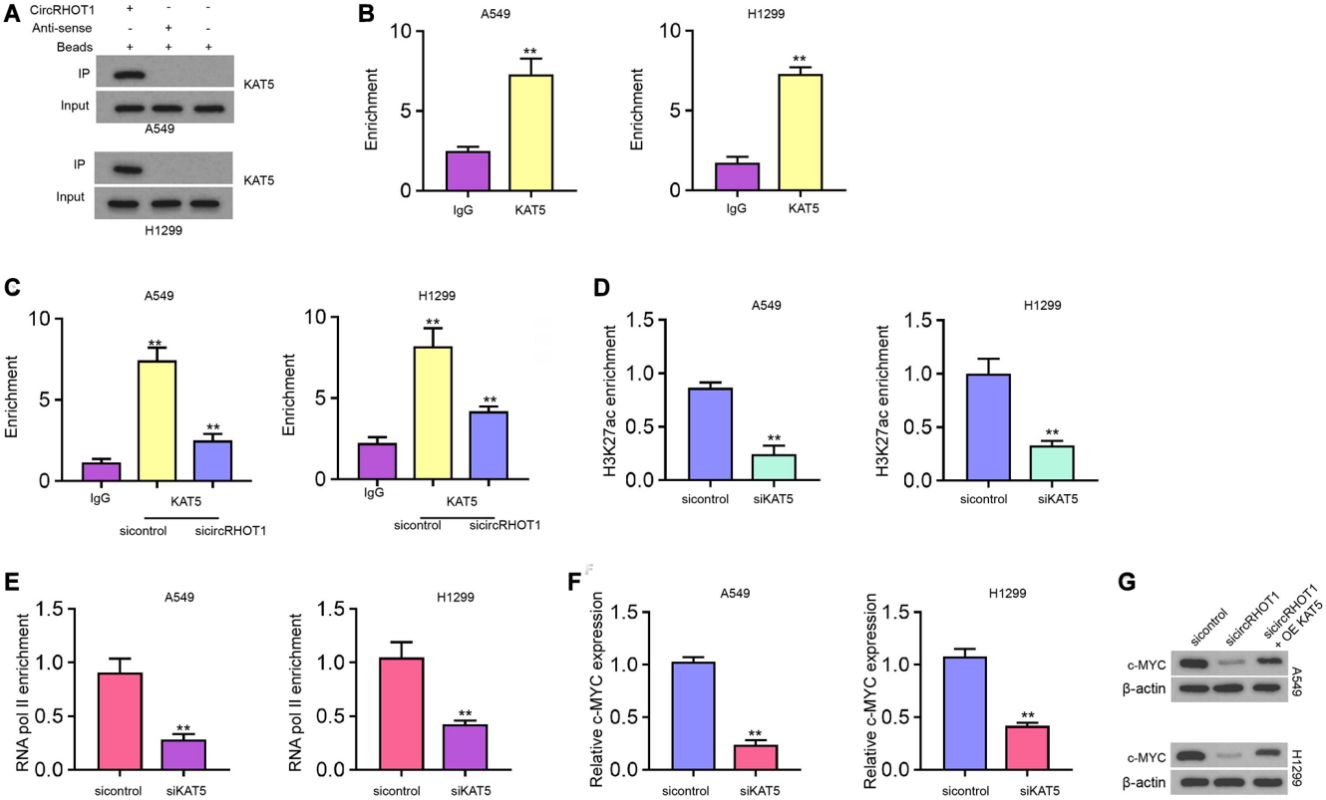
**CircRHOT1 epigenetically regulates c-MYC expression by recruiting KAT5 in NSCLC cells.** (**A**) The interaction of circRHOT1 and KAT5 was measured by RNA pull down in A549 and H1299 cells. (**B**) The enrichment of KAT5 on c-MYC promoter was analyzed by ChIP. (**C**) The enrichment of KAT5 on c-MYC promoter was analyzed by ChIP in A549 and H1299 cells treated with circRHOT1 siRNA. (**D**) The enrichment of H3K27ac on c-MYC promoter was analyzed by ChIP in A549 and H1299 cells treated with KAT5 siRNA. (**E**) The enrichment of RNA polymerase II on c-MYC promoter was analyzed by ChIP in A549 and H1299 cells treated with KAT5 siRNA. (**F**) The mRNA expression of c-MYC was tested by qPCR in A549 and H1299 cells treated with KAT5 siRNA. (**G**) The protein levels of c-MYC were determined by Western blot analysis in A549 and H1299 cells co-treated with circRHOT1 siRNA and KAT5 overexpression vector. mean ± SD, ^**^P < 0.01.

Next, we further explored the underlying mechanism of circRHOT1-mediated epigenetic modification of c-MYC. RNA pull down analysis showed that circRHOT1 was able to directly interact with acetyltransferase KAT5 in H1299 and A549 cells (Figure 5A). Meanwhile, we observed that KAT5 could bind to c-MYC promoter in H1299 and A549 cells (Figure 5B). The silencing of circRHOT1 significantly impaired the enrichment of KAT5 on the c-MYC promoter in H1299 and A549 cells (Figure 5C). Moreover, the depletion of KAT5 reduced the enrichment of H3K27ac and RNA polymerase II on the c-MYC promoter (Figure 5D and 5E). Consistently, the inhibition of KAT5 reduced c-MYC expression in H1299 and A549 cells (Figure 5F). The depletion of circRHOT1 down-regulated c-MYC expression, while KAT5 overexpression could reverse this down-regulation in H1299 and A549 cells (Figure 5G).

### CircRHOT1/c-MYC signaling contributes NSCLC cell proliferation *in vitro* and *in vivo*

**Figure 6:**
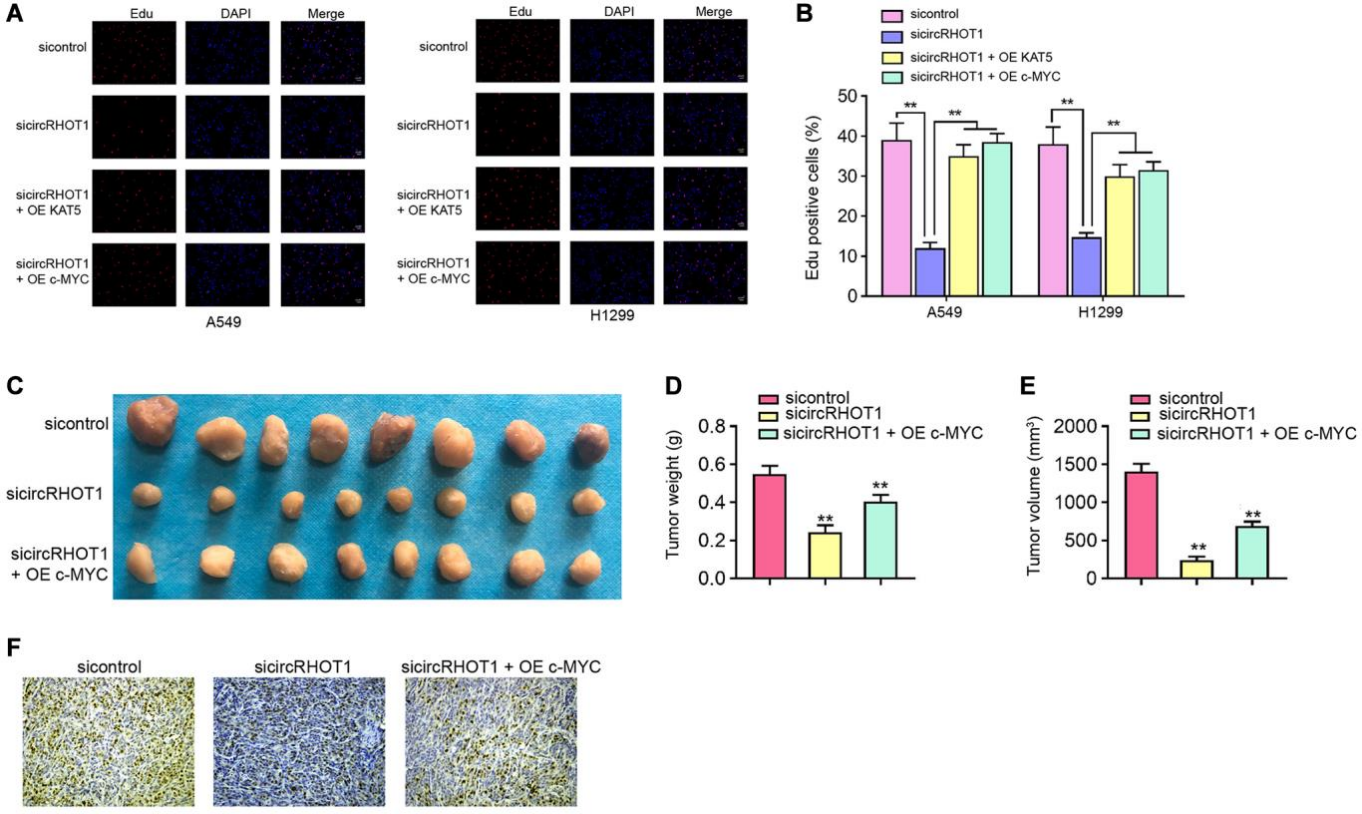
**CircRHOT1/c-MYC signaling contributes NSCLC cell proliferation *in vitro and in vivo*.** (**A** and **B**) The A549 and H1299 cells were treated with circRHOT1 siRNA or co-treated with circRHOT1 siRNA and KAT5 overexpression vector or c-MYC overexpression vector. The cell survival was detected by Edu assays. (**C**–**F**) The nude mice (*n* = 5) were injected with A549 cells treated with circRHOT1 siRNA or co-treated with circRHOT1 siRNA and c-MYC overexpression vector. The tumor image (**C**), tumor weight (**D**), and tumor volume (**E**) were shown. (**F**) The Ki-67 levels were analyzed by IHC staining. mean ± SD, ^**^P < 0.01.

Next, we validated the effect of circRHOT1/c-MYC signaling on NSCLC cell proliferation *in vitro a*nd *in vivo*. Edu assays showed that the silencing of circRHOT1 reduced cell proliferation of H1299 and A549 cells, in which the overexpression of c-MYC or KAT5 could rescued the reduction in the cells (Figure 6A and 6B). Tumorigenicity analysis in nude mice further proved that the depletion of circRHOT1 attenuated A549 cell growth *in vivo*, while the overexpression of c-MYC was able to reverse this effect (Figure 6C–6E). The levels of ki-67 was decreased by circRHOT1 knockdown and c-MYC overexpression rescued the levels in the tumor tissues (Figure 6F).

## DISCUSSION

NSCLC one of the most prevalent and severe malignancies in the world. The molecular mechanisms of NSCLC are poor understood, limiting the development of diagnostic biomarkers and targeted therapies. CircRNAs have been identified as a type of critical regulator in cancer progression. In this study, we identities the epigenetic regulation function of circRHOT1 in promoting NSCLC cell proliferation.

Previous studies have reported the correlation circRHOT1 with cancer development. It has been reported that circRHOT1 enhances the progression of hepatocellular carcinoma by inducing NR2F6 expression [21]. CircRHOT1 is involved in the modulation of cell invasion, apoptosis, and proliferation by targeting miR-125a-3p in pancreatic cancer cells [11]. CircRHOT1 is increased and contributes to cell invasion and proliferation of pancreatic cancer cells [12]. Our data showed that circRHOT1 was elevated in clinical NSCLC samples and NSCLC cells lines. It implies the association of circRHOT1 with NSCLC in clinical and experimental conditions. In this study, we just investigated the effect of circRHOT1 on NSCLC at experimental condition and the clinical significance of circRHOT1 in NSCLC is needed to explore in future investigation. Meanwhile, some circular RNAs are identified in the regulation of NSCLC development. It has been reported that circular RNA circFGFR1 contributes to anti-PD-1 resistance and progression of NSCLC by sponging miR-381-3p [22]. Circular RNA circHIPK3 regulates autophagy in NSCLC cells by MIR124-3p-STAT3/PRKAA/AMPKα signaling [23]. Circular RNA circ-CPA4/let-7/PD-L1 signaling modulates immune evasion, drug resistance, stemness, cell growth in NSCLC [24]. Circular RNA circSATB2 induces NSCLC progression [25]. In this study, we found that circRHOT1 contributes to cell proliferation of NSCLC cell *in vitro* and *in vivo*. It indicates that circRHOT1 is a crucial regulator of NSCLC development, providing another evidence of the significant roles of circular RNA in NSCLC.

Moreover, it has been reported that CDK5 reduces the tumor inhibitory impact of BIN1 by regulating c-MYC Ser-62 phosphorylation in NSCLC [26]. C-myc/miR-150/EPG5 signaling regulates the autophagy dysfunction and contributes to NSCLC development [27]. PGE2-stimulated c-Myc expression induces NSCLC resistance to apoptosis [28]. Our data showed that circRHOT1 enhanced c-Myc expression in NSCLC cells and c-Myc overexpression reversed circRHOT1 depletion-inhibited NSCLC cell proliferation. It indicates that circRHOT1 may contribute to NSCLC cell proliferation by inducing c-Myc. c-My may just one of the downstream factors of circRHOT1 and more targets of circRHOT1 and their relationship in NSCLC are needed further investigate. Moreover, it has been reported that KAT5 recruited by FosB contributes to metastasis and growth of papillary thyroid cancer [29]. KAT5 enhances metastasis and invasion by stabilizing c-MYC in thyroid cancer [20]. In the present investigation, we found that circRHOT1 epigenetically enhances c-MYC expression by recruiting KAT5 in NSCLC cells. The overexpression of KAT5 reversed circRHOT1 depletion-inhibited NSCLC cell proliferation *in vitro*. It provides an innovative correlation of KAT5 with circRHOT1 and the epigenetic function of circRHOT1 by regulating KAT5. Meanwhile, the chemo-reagents or specific inhibitors targeting circRHOT1 or KAT5 should be explored by more investigations.

In summary, we concluded that circRHOT1 contributed to pathogenesis of NSCLC by epigenetically enhancing c-MYC expression through recruiting KAT5. CircRHOT1 and KAT5 may be used as the potential targets for NSCLC therapy.
